# Longevity of Women

**Published:** 1881-04

**Authors:** 


LONGEVITY OF WOMEN.—The recent European statistical returns of the population of Europe have supplied the statistical department at Vienna with the means of making an interesting study as regards longevity. It results from this, that of 102,831 individuals who had exceeded the age of ninety years, 60,303 were women, and 42,528 men. The greater longevity of women is exhibited also by the greater chances of women of attaining or exceeding the hundredth year. Thus, in Italy there are found 421 female centenarians for 141 male centenarians, and in Austria 229 women for 183 men. In Austria there are 1,508,359 sexagenarians, or 7.5 per cent. of the total population.—*Medical and Surgical Reporter.*

